# First Report of Colonies of Sylvatic *Triatoma infestans* (Hemiptera: Reduviidae) in the Paraguayan Chaco, Using a Trained Dog

**DOI:** 10.1371/journal.pntd.0001026

**Published:** 2011-05-03

**Authors:** Miriam Rolón, María Celeste Vega, Fabiola Román, Ana Gómez, Antonieta Rojas de Arias

**Affiliations:** Centro para el Desarrollo de la Investigación Científica (CEDIC/Díaz Gill Medicina Laboratorial/Fundación Moisés Bertoni), Asunción, Paraguay; IRD/CIRDES, Burkina Faso

## Abstract

In the Gran Chaco region, control of *Triatoma infestans* has been limited by persistent domestic infestations despite the efforts of the Vector Control Services. In Paraguay, this region is the highest endemic area in the country, showing high levels of indoor and outdoor infestation. Although sylvatic *T. infestans* have been found in the Bolivian and Argentine Chaco, similar searches for sylvatic populations of this species in Paraguay had been unsuccessful over the last 20 years. Here we present a new approach to detecting sylvatic Triatominae, using a trained dog, which has successfully confirmed sylvatic populations of *T. infestans* and other triatomine species in Paraguay. A total of 22 specimens corresponding to dark morph forms of *T. infestans* were collected, and 14 were confirmed as *T. infestans* by the mitochondrial cytochrome B gene analysis. Through this analysis, one of which were previously reported and a second that was a new haplotype. Triatomines were captured from amongst vegetation such as dry branches and hollows trees of different species such *Aspidosperma quebracho-blanco*, *Bulnesia sarmientoi* and *Stetsonia coryne*. The colonies found have been small and without apparent infection with *Trypanosoma cruzi*. During the study, *Triatoma sordida* and *Triatoma guasayana* have also been found in ecotopes close to those of *T. infestans*.

## Author Summary

Confirmation of sylvatic colonies of *Triatoma infestans* has a significant connotation for Paraguay. Prior to our findings, we believed this vector —unlike in other regions of the Gran Chaco—was living exclusively in domestic and peridomestic habitats. We never considered the possibility of sylvatic species re-infesting domiciliary dwellings. After this discovery, the frame of transmission dynamics of *Trypanosoma cruzi* in the Paraguayan Chaco proposes new research perspectives. This also opens the door to promote knowledge regarding potential genetic flows between different *T. infestans* populations, reservoirs associated with their colonies, as well as their impact over control actions. Fieldwork for wild species identification is difficult and often unsuccessful, we used several techniques and tools, proven by others such as light traps, and mouse-baited sticky traps however, the triatomine collection in our study area was scarce or null. Incorporating a trained dog – NERO – to our work team has been a highly successful and productive initiative. The surprising ability NERO has shown will enable us to provide specific data regarding the still unknown wild ecotopes of *T. infestans*, as well as the potential use of trained dogs as a community surveillance tool of triatomine species considered particularly important for public health.

## Introduction

*Triatoma infestans* (Hemiptera, Reduviidae) is the main vector of Chagas disease (American trypanosomiasis) in the Southern Cone of Latin America. Through the Southern Cone Initiative against Chagas disease, vectorial transmission to humans has been interrupted in Chile, Uruguay and Brazil, but Argentina and Paraguay have achieved this only in some regions [1]. In the Gran Chaco region, comprising parts of Argentina, Bolivia and Paraguay, control of the vectors has been limited due to the persistence of domestic infestations despite the efforts of the Vector Control Services in these countries [2], [3].

Studies conducted since the 1970s have shown high levels of indoor infestation of *T. infestans* in the Paraguayan Chaco, characterizing this region as the highest endemic area in the country [4]–[8]. However, sylvatic populations of this vector have only occasionally been reported in Paraguay [9] although nymphs of *T. infestans* were recently reported amongst vegetation near indigenous dwellings [10]. By contrast, sylvatic *T. infestans* have been more frequently reported from the Andean valleys of Cochabamba and La Paz in Bolivia, and also in the Bolivian Chaco [11]–[13] and the Argentine Chaco [9], [14]. The finding of dark morph (DM) *T. infestans* in parrot nests in Argentina [14], and the finding of extensive new foci of sylvatic triatomine populations in Bolivia [15] encouraged the intense search in the Paraguayan Chaco region, but the search for this species using light traps and manual checking of fallen trees and burrows had been unsuccessful. We report here a novel approach using a trained dog, which has revealed several sylvatic populations of *T. infestans* in the Paraguayan Chaco.

Domestic dogs (*Canis familiaris*) are used by humans to locate a range of substances because of their superior olfactory acuity. Their area of olfactory epithelium (18 to 150 cm^2^) [16] is much greater than that of humans (3 cm^2^) [17]. They are widely used to detect non-biological (explosives, chemical contaminants, illegal drugs) and biological scents (human odours, animal scents) and have an important role in conservation [18]. Dogs have been trained for search and rescue of missing people [19], to search for brown tree snakes [20], insects that damage plants [21], birds [22], egg masses of gypsy moths [23], subterranean termites [24], screwworm-infested wounds [25], catfish off-flavour compounds [26], animal scat detection [27] and microbial organisms such as rot fungi, building moulds, and bacteria [28]. However, as far as we know, there are no previous attempts to train dogs to detect triatomine bugs.

Triatominae produce volatile compounds, which seem to play a role in their defense and alarm processes, as well as in sexual communication and mating. The Brindley's glands, present in adult Triatominae, seem mainly to secrete isobutyric acid – believed to be involved in defense against predators [29], [30]. The metasternal glands, also present in adults, have been associated with sexual communication, and some highly volatile ketones (3-pentanone) and alcohols that are emitted by adults during mating have been identified [29], [30]. Moreover, the nymphs do not have Brindley's glands, metasternal glands, or dorsal abdominal glands [31]. The bug faeces are also a source of attractants [32] and both adults and nymphs respond to faeces from different species [33]–[37]. The compounds most commonly found in fresh faeces are ammonia and uric acid, and other compounds such as o-aminoacetophenone, 4-methylquinazoline, 2,4-dimethylquinazoline, and 2-pyrolidinone [37], [38].

Based on the possibility of detecting bugs by means of their odours we have implemented a new method in which we use a trained dog to search for triatomines. This has enabled us to find sylvatic *T. infestans* in the region of the Paraguayan Chaco through a quick, easy and low-cost procedure.

## Materials and Methods

### Ethics Statement

The study in the indigenous communities was approved by the local Ethical Committee of the Fundación Moisés Bertoni (IDRC Grant No. 103696-009- Revision 07/27/2007) and CEDIQUIFA (Approved 02/18/2008) (Argentina). Following local indigenous conventions for the approval of research in their communities, the local leaders of the villages of 12 de Junio and 10 Leguas were informed of the study objectives prior to commencing the study and they signed an informed consent form on behalf of the members of the community. This village-level consent process was approved by both ethics committees.

The use and handling of animals in this study was approved by Fundación Moisés Bertoni (Grant No. 103696-009-Addendum 05/03/2010) and the animal care and facilities supporting this activity was maintained according to the standards of the Council for International Organizations of Medical Sciences (CIOMS, 1985) [39].

### Area of study

**Figure 1:**
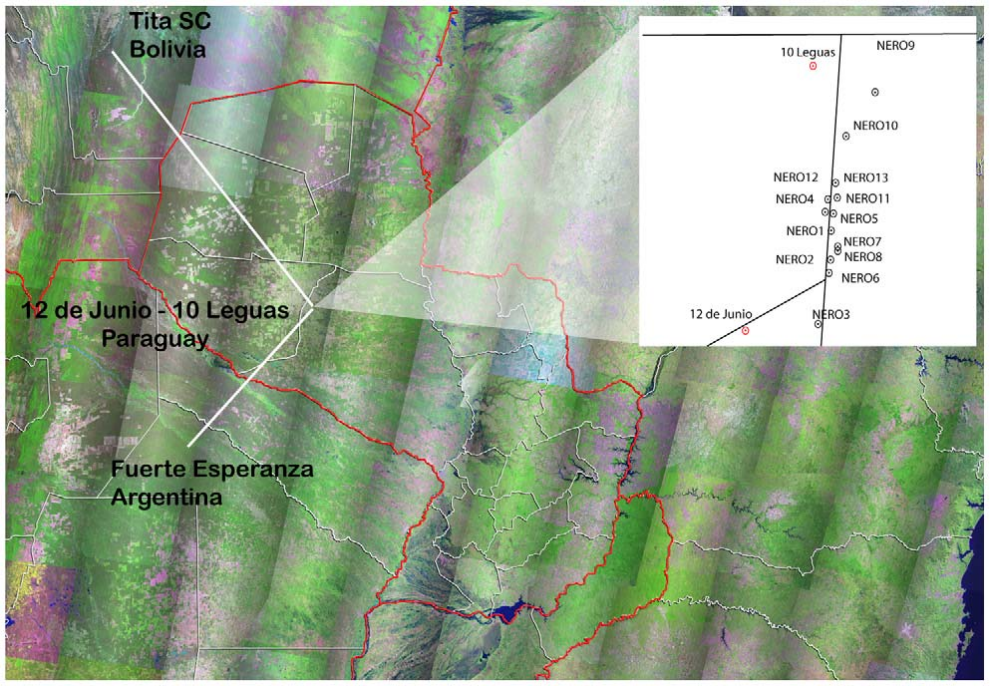
United State Geological Survey (USGS) aerial photo images of positive sites for wild populations of *T. infestans* in the Gran Chaco.

Within the framework of an entomological surveillance study of indigenous communities, sylvatic triatomines were sought within the peridomicile of the indigenous communities of 12 de Junio and 10 Leguas in the Department of Presidente Hayes (Figure 1). The surrounding area represents typical xeromorphic Chaco woodland, characterized by species such as *Aspidosperma quebracho-blanco*, *Schinopsis quebrachocolorado*, *Bulnesia sarmientoi*, *Prosopis nigra*, *Schinopsis balansae*, *Calycophyllum multiflorum y Stetsonia coryne* [40], [41]. The climate in this part of the Chaco is characterized by extreme summer heat and mild winters. Temperature extremes range from 45° C in spring and summer to −7°C in winter. Windspeed averages 3.3 meters/second (11.9 km/h) that increases up to 3.9 m/s (14.0 km/h) in winter [42].

For this study geo-referenced points were identified using a GPS (GARMIN Etrex Legend) during field trips.

### Triatomine capture method and dog training

**Figure 2:**
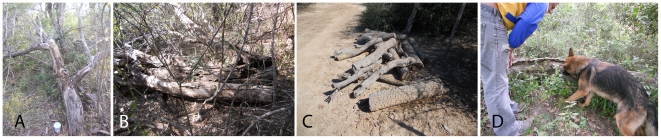
Different capture areas: *Tabara major nes*t in fallen *palo santo* tree (A), rodent burrows in fallen *quebracho blanco* tree (B), firewood of *quebracho blanco* (C), NERO and his trainer in the Chaco sylvatic area (D).

Triatomines were manually captured in demarcated areas during daylight hours with the help of NERO, a 9 month-old gray German Shepherd male dog (Figure 2D). NERO had basic obedience training and was further trained to locate triatomines by an experienced dog trainer. The trainer used live, laboratory-reared, uninfected male and female adult bugs throughout the training process. The specimens were placed individually in plastic containers closed with gauze, with paper as a substrate.

Training was carried out in the trainer's home using the method outlined by the United States Customs Service [43]. First the living triatomines were presented to the dog to stimulate the dog's olfactory memory before being hidden somewhere in a house, and the dog was told to “search”. After daily training sessions for 3 weeks, the triatomines were no longer presented to the dog at the beginning of the session, and the dog was asked to “search” for hidden bug samples. In the third phase, several samples were hidden around the house simultaneously. The dog's ability to locate different intensities of odor was tested by hiding samples of several bugs at some sites and single bugs at other sites. Tasks with no positive samples were included as well. When the dog found the sample, he would sit at attention next to the sample and look at his trainer. Small pieces of sausage were used as rewards. The training took a total of 3 months.

In the field, the dog was accompanied by his trainer and a field team made up of three or four biologists. Every time the dog made the appropriate signal the field team made a thorough revision of the area looking for triatomines. The collection of triatomines was carried out 5 times during the months of May to August 2010.

### Laboratory procedures

The place and characteristics where triatomines were found were geo-referenced and noted with the climatic characteristics of the days when captures were carried out. Specimens were placed together in plastic cups with paper as a substrate, coded according to capture sites, and transported live to the laboratory where they were classified by species, sex, and stage following standard taxonomic keys [31]. Faecal matter expressed from each specimen was also checked microscopically at 400× for possible trypanosome infection. Specimens were then preserved in 70% ethanol for subsequent DNA extraction from legs.

### Molecular identification of specimens

For DNA extraction, four legs from each specimen were ground to a fine powder in the presence of liquid nitrogen, mixed with 1 mL of lysis buffer, and incubated overnight at 37°C [44]. DNA was extracted sequentially with phenol, phenol-chloroform-isoamyl alcohol, and chloroform-isoamyl alcohol, and precipitated with ethanol in 0.3 M sodium acetate [45]. The mitochondrial cytochrome B gene was targeted for amplification as described by Lyman et al [46] and a frangment of 415 bp (primer regions not included) with no insertions or deletions was considered in the analysis. PCR products were sequenced directly and in both directions. Sequences from sylvatic bugs were compared with GenBank *Triatoma* spp. sequences by Blast analysis with Genbank default parameters.

### Single-blind canine scent-testing experiments

To determine if the dog was able to differentiate between nymph and adult triatomines, laboratory-reared 3^rd^ and 5^th^ stage *T. infestans* were placed in plastic containers and hidden for the dog to search for them. Similarly, two trials were conducted to assess which triatomine odours the dog could detect. In the first, a plastic vial containing a filter paper impregnated with 50 uL of commercial isobutyric acid (MERCK) was hidden. The second trial used papers impregnated with fresh or dried faeces from adult and nymph stage *T. infestans*. Each of these trials was done on two occasions in the trainer's house.

## Results

A total of 70 triatomines was collected during 5 field trips with NERO. All specimens were captured alive from vegetation such as dry branches, hollow or standing trees of different species like *quebracho blanco (Aspidosperma quebracho-blanco)*, verawood [better known by its spanish name *palo santo*] *(Bulnesia sarmientoi)* and dried cactus *(Stetsonia coryne)*. In the case of quebrachos, the bugs were found inside hollow dry branches, while in palo santo they were captured from the cortex. Triatomines were also found in a *Tabara major* nest in a fallen *quebracho blanco* tree (Figure 2A), in rodent burrows inside a fallen *palo santo* tree (Figure 2B) and in piles of *quebracho blanco* branches cut for firewood (Figure 2C). The house nearest to capture sites was located 408 meters from the town of 10 Leguas, while the distances from capture sites to the nearest community averaged 2.8±1 km (Figure 1).

Of the 70 bugs collected, 22 specimens (16 adults and 6 nymphs) corresponded to dark morph (DM) forms of *T. infestans* (Table 1). Purified DNA from 14 of these successfully amplified the target cyt-b fragment, resulting in two haplotypes that differed in 4 synonymous substitutions. The Blast analysis with sequences available in GenBank confirmed them as *T. infestans*. One of the haplotypes presented 100% similarity to already published sequences (accession numbers AY062165.1 and EF639038.1). The other haplotype is now deposited in Genbank under accession number HQ848648.

**Table 1:** Distribution of sylvatic *Triatoma infestans* captured in different ecotopes in the Paraguayan Chaco.

Nearest community	Localization code	Latitude	Longitude	Height (meters)	Capture location (distance from community)	Date of capture	Num. of triatomines and stages
12 de Junio	NERO1	22°54′44,23″S	59°52′33,73″W	131	Dry branch of *quebracho blanco* (3345 m)	18/06/2010	♀
	NERO2	22°55′8,29″S	59°52′34,43″W	130	Dry branches of *palo santo* (2763 m)	18/06/2010	♀ , NV
	NERO3	22°55′44,06″S	59°52′56,65″W	129	Fallen and dry *palo santo* tree (1593 m)	18/06/2010	♂
	NERO4	22°54′27,96″S	59°52′32,53″W	131	Bark of standing *quebracho blanco* (3759 m)	06/08/2010	♀ , NV
	NERO5	22°54′31,24″S	59°52′30,85″W	129	Fallen and dry *palo santo* tree (3708 m)	06/08/2010	♂ , NV
	NERO6	22°55′16,92″S	59°52′35,63″W	131	*Quebracho blanco* fire wood (2580 m)	07/08/2010	♀ , NV
	NERO7	22°55′00,32″S	59°52′32,78″W	130	Rodent burrow in fallen *quebracho blanco* (2962 m)	07/08/2010	♀
	NERO8	22°55′00,81″S	59°52′32,79″W	131	Bark of fallen *quebracho blanco* (2951 m)	07/08/2010	♂
10 Leguas	NERO9	22°52′20,35″S	59°52′4,20″W	130	Dry branches of *quebracho blanco* (408 m)	01/06/2010	3 ♂, ♀
	NERO10	22°53′28,10″S	59°52′19,74″W	129	Nest of *Tabara major* in fallen *palo santo* tree (1952 m)	14/07/2010	♂
	NERO11	22°54′23,48″S	59°52′31,42″W	131	Dry fallen cactus tree (3671 m)	06/08/2010	♀ , NIII
	NERO12	22°54′26,74″S	59°52′31,50″W	131	Dry branches of *quebracho blanco* (3751 m)	06/08/2010	♀
	NERO13	22°54′16,88″S	59°52′27,28″W	131	Dry branches of fallen *quebracho blanco* (3438 m)	07/08/2010	♀ , NV

The remaining bugs comprised 18 specimens of *Triatoma guasayana*, and 30 specimens of *Triatoma sordida* (Table 2). There was a predominance of *T. sordida* in relation to *T. infestans*, and although both species were found in the same period of time they were never found sharing the same habitat. None of the specimens of any species examined under the microscope appeared to be infected with trypanosomes.

**Table 2:** Distribution of sylvatic *Triatoma sordida* and *Triatoma guasayana* captured in different ecotopes in the Paraguayan Chaco.

Nearest community	Localization code	Latitude	Longitude	Height (meters)	Capture location (distance from community)	Date of capture	Num. of triatomines and stages
*T. sordida*							
12 de junio	SOR1	22°55′54,38″S	59°53′46,39″W	131	Nest in fallen *palo santo* tree (500 m)	31/05/2010	NIII, 4 NV
	SOR2	22°55′53,95″S	59°53′45,51″W	130	Fallen and dry *palo santo* tree (493 m)	31/05/2010	2 NV
	SOR3	22°55′54,12″S	59°53′35,51″W	129	*Quebracho blanco* fire wood (561 m)	01/06/2010	NV
	SOR4	22°55′24,44″S	59°52′32,97″W	131	Dry branches of *quebracho blanco* (2495 m)	01/06/2010	NIV
	SOR5	22°55′22,69″S	59°52′32,30″W	129	Dry branches of *quebracho blanco* (2544 m)	01/06/2010	NV
	SOR6	22°54′31,39″S	59°52′29,42″W	130	Fallen and dry *palo santo* tree (3733 m)	06/08/2010	♀
10 leguas	SOR7	22°52′21,45″S	59°52′9,26″W	135	Fallen and dry *palo santo* tree (260 m)	17/05/2010	2 NIII, 1 NIV
	SOR8	22°52′20,60″S	59°47′54,60″W	131	Fallen parrot nest (7501 m)	17/05/2010	♂
	SOR9	22°53′41,35″S	59°53′2,90″W	127	Dry branch of *quebracho blanco* (2681 m)	18/05/2010	2 NIV
	SOR10	22°53′41,46″S	59°53′2,21″W	126	Dry branch of *quebracho blanco* (2700 m)	18/05/2010	NIV
	SOR11	22°53′14,90″S	59°52′23,31″W	130	Dry fallen cactus tree (1507 m)	18/05/2010	NIII, NIV
	SOR12	22°53′14,15″S	59°52′18,09″W	128	Dry fallen cactus tree (1504 m)	19/05/2010	NIV
	SOR13	22°52′23,32″S	59°52′9,55″W	127	*Quebracho blanco* fire wood (230 m)	01/06/2010	NIV
	SOR14	22°52′22,13″S	59°52′8,39″W	122	Dry branch of *quebracho blanco* (274 m)	01/06/2010	NV
	SOR15	22°53′38,43″S	59°52′39,51″W	131	Fallen and dry *palo santo* tree (2342 m)	17/06/2010	NV
	SOR16	22°53′47,45″S	59°52′25,92″W	134	Dry branches of *quebracho blanco* (2542 m)	17/06/2010	NV
	SOR17	22°53′48,33″S	59°52′24,87″ W	129	Bark of standing *quebracho blanco* (2560 m)	17/06/2010	NV, NIV
	SOR18	22°54′22,61″S	59°52′30,06″W	129	Bark of standing *quebracho blanco* (3615 m)	18/06/2010	NII, NIII, NV
*T. guasayana*							
12 de junio	GUA1	22°54′21,72″S	59°52′30,72″W	131	Fallen and dry *palo santo* tree (3890 m)	06/08/2010	♂, 3 NIII
	GUA2	22°54′31,39″S	59°52′29,42″W	130	Stump of *palo santo* (3733 m)	06/08/2010	2 ♂
	GUA3	22°55′38,83″S	59°53′8,70″W	130	Dry branch of *quebracho blanco* (1417 m)	07/08/2010	2 ♀
10 leguas	GUA4	22°52′21,83″S	59°52′09,34″W	135	Fallen and dry *palo santo* tree (258 m)	19/05/2010	1 ♂
	GUA5	22°54′15,60″S	59°52′24,65″W	129	Dry branch of *quebracho blanco* (3388 m)	06/08/2010	♂, ♀
	GUA6	22°54′15,85″S	59°52′25,14″W	130	Dry fallen cactus tree (3409 m)	07/08/2010	3 ♀
	GUA7	22°54′15,85″S	59°52′25,14″W	130	Fallen and dry *quebracho blanco* tree (3444 m)	07/08/2010	4 ♀

Following the discovery of triatomine colonies in the forested areas of the Chaco we attempted further trials to see if the dog was capable of identifying nymphs and adults independently, and what specific scent the dog was detecting. The dog was exposed to nymphs, fresh and dry bug faeces, and isobutyric acid, in independent experiments. The dog consistently marked the location of the nymphs, but did not find the fresh or dry faeces on any occassion. When the dog was exposed to a flask containing isobutyric acid the dog was able to locate the flask, but did not clearly indicate the location as when finding a live triatomine bug.

## Discussion

This is the first finding of sylvatic dark-morph (DM) *T. infestans* colonies in the Central Chaco of Paraguay. There are three previous reports of catches of *T. infestans* in sylvatic areas of Paraguay, but these were attributed to peri-domiciliary populations [47]–[49], [10].

Prior to this study, we had repeatedly attempted to find sylvatic *T. infestans* in the same region using light traps [11] and Noireau traps [50]. From this, a single *T. infestans* adult of non-melanic domestic phenotype was caught in a light trap located near a house (and consequently assumed to have flown from the house). By contrast, the use of a trained dog has yielded confirmation of the existence of sylvatic *T. infestans* in the region, with the additional benefit that the bugs can be located in their habitat – whereas the use of traps requires the bugs to leave their habitats.

The dog detected sylvatic *T.infestans* mainly in hollow fallen trees. Empty burrows and traces of rodent droppings were always found near these trees, which suggests that wild rodents are an important sylvatic host, as also indicated by observations in the Bolivian Chaco [51]. All the findings of *T. infestans* by the dog included adult bugs, but some of the findings of *T. sordida* colonies appeared to contain only nymphal stages, which suggests that the dog is able to find both adults and nymphs. In the scent-testing experiments, the dog detected nymphs of *T. infestans* without having had previous contact with them, and did not appear to recognise isobutyric acid (which is secreted only by adult Triatominae) in the same way as finding the bugs themselves. This suggests that the odour recognition stimulus for the dog is common to nymphs and adults, and does not correspond to Brindley's or metasternal glands which are absent or non-functional in nymphs [31]. Similarly, the dog detected the presence of *T. sordida* and *T. guasayana* as well as *T. infestans*, suggesting that the odour recognition stimulus is common to different species of Triatominae, but the dog did not detect samples of fresh or dried bug faeces, suggesting that these are not the source of the odour recognition stimulus.

**Figure 3:**
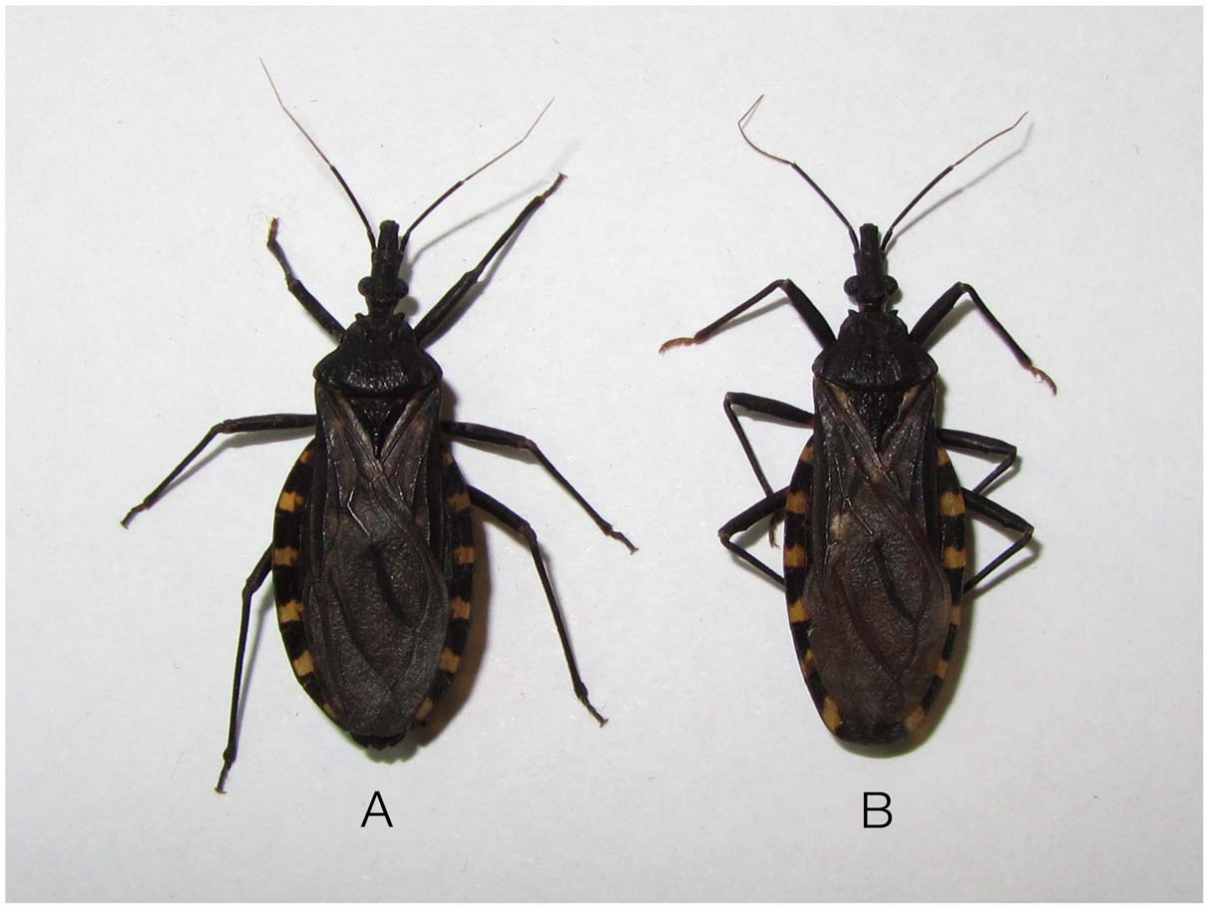
Female (A) and male (B) *T. infestans* dark morph captured from sylvatic areas in the paraguayan Chaco.

The color of the sylvatic adult DM *T. infestans* (Figure 3) was clearly different from the domestic forms collected over a 12-month period in the communities of 12 de Junio and 10 Leguas. Sylvatic specimens were darker and larger than the domestic ones and had similar characteristics to those described as sylvatic specimens in Argentina [14] but slightly different to those found in Bolivia [52] especially with regard to the yellow spots of the connexivum, which were smaller in Paraguayan DM forms.

The distances between our capture sites and those conducted in Tita, Chaco Boreal [50] and Fuerte Esperanza, Argentina [14] are 559.8 km and 348.9 km, respectively, which could indicate a wide distribution of these melanic forms (Figure 1). However, we noted that adults and nymphs of the dark morph (DM) type take a lighter brown color when held in the laboratory for several weeks, especially on the pronotum. It may be, therefore, that a melanic coloration is an adaptive characteristic for the sylvatic habitat [14]. Significantly, unlike the great distances between DM forms and domestic populations in the Bolivian Chaco, we have found DM colonies at distances not exceeding 3 km from infested houses, which could allow us to assess gene flow between sylvatic and domestic forms in order to assess if the sylvatic populations occur independently or as derivatives of the domestic ones [53].

The remaining sylvatic species caught, *T. sordida* and *T. guasayana*, are abundant in the Chaco. Several studies described these species in sylvatic areas, and often from peridomestic habitats such as chicken coops [54]–[56] as well as in cactus and bromeliad plants [51]. Both species have been found sharing niches in the Paraguayan Chaco, particularly in bird nests in palm trees [57]. Also, adults of these two species are sometimes found in houses where *T. infestans* has been eliminated by insecticide spraying (data not shown). In the Chaco, *T. guasayana* flies readily in search for food [10] and is considered a secondary vector of Chagas disease in parts of Argentina [55].

In a previous study carried out in 1993 by one of the authors, a group of Ayoreo indigenous communities with different sedentary levels were evaluated. Due to their semi-nomadic characteristics, Chovoreca and Gesudi groups from the same Chaco region, lived in temporary shelters which were infested only with *T. sordida*. Strikingly, these groups did not recognize *T. infestans* when prompted. However, in the already sedentary group of Campo Loro, where houses are built with mud and straw (similar to many Paraguayan rural peasant dwellings), there were substantial domestic and peridomestic infestations with *T. infestans* [54]. The presence of this vector in permanent dwellings rather than temporary ones, could make the case for the hypothesis of *T. infestans* dispersal due to human migration from the Andean valleys of Bolivia and the subsequent re-colonization of wildlife habitat by domestic species [53].

But although sylvatic DM forms of *T. infestans* have now been described from areas of Argentina, Bolivia and Paraguay, there are still large areas in the Gran Chaco that have not been evaluated in the search for sylvatic populations. The dry Chaco biome is a large geographical area that includes the three countries, which is why greater efforts should be made to determine the extent of *T. infestans* distribution and the role of DM forms in the reinfestation of houses in the region. Using trained dogs to find triatomines is a method that reduces sample collection bias and decreases the time spent searching for sylvatic specimens, which are invariably present in very low densities and dispersed over wide areas. In addition, if acceptable to local communities, the use of trained dogs may also facilitate entomological surveillance for domestic infestations of Triatominae, especially in monitoring houses for early signs of reinfestation following control interventions.

### Accession Number

DNA sequence of 660 bp of the new haplotype including the cytochrome B gene was deposited in GenBank under accession number HQ848648. This sequence was obtained using the primers described by Monteiro et al [58].

## References

[1] Schofield CJ, Jannin J, Salvatella R (2006). The future of Chagas disease control.. Trends Parasitol.

[2] Gürtler RE, Kitron U, Cecere MC, Segura EL, Cohen JE (2007). Sustainable vector control and management of Chagas disease in the Gran Chaco, Argentina.. Proc Natl Acad Sci USA.

[3] Gürtler RE (2009). Sustainability of vector control strategies in the Gran Chaco Region: Current challenges and possible approach.. Mem Inst Oswaldo Cruz.

[4] Canese A, Canese J (1976). Estudio de los vectores de la enfermedad de Chagas en varias regiones del Paraguay.. Rev Paraguaya Microbiol.

[5] Canese J, Brice E (1979). Elevado índice de serología positiva para enfermedad de Chagas en el Chaco Paraguayo (XV Dpto Pte Hayes).. Rev Paraguaya Microbiol.

[6] Rojas de Arias A, Monzón MI, Velázquez Guillén G, Arrúa Torreani N (1984). Seroepidemiología de la enfermedad de Chagas en el Paraguay.. Bull Pan Am Health Organ.

[7] Rojas de Arias A Chagas disease in Paraguay..

[8] Rojas de Arias A, Ferro E, Ferreira ME, Simancas L (1999). Chagas disease vector control through different interventions modalities in endemic localities of Paraguay.. Bull World Health Organ.

[9] Abalos JW, Wygodzinsky P (1951). Las Triatominae Argentinas (Reduviidae, Hemiptera)..

[10] Yeo M, Acosta N, Llewellyn M, Sánchez H, Adamson S (2005). Origins of Chagas disease: *Didelphis* species are natural hosts of *Trypanosoma cruzi* I and armadillos hosts of *Trypanosoma cruzi* II, including hybrids.. Int J Parasitol.

[11] Noireau F, Flores R, Gutierrez T, Dujardin JP (1997). Detection of wild dark morphs of *Triatoma infestans* in the Bolivian Chaco.. Mem Inst Oswaldo Cruz.

[12] Cortez MR, Pinho AP, Cuervo P, Alfaro F, Solano M (2006). *Trypanosoma cruzi* (Kinetoplastida Trypanosomatidae): ecology of the transmission cycle in the wild environment of the Andean valley of Cochabamba, Bolivia .. Exp Parasitol.

[13] Cortez MR, Emperaire L, Piccinali RV, Gürtler RE, Torrico F (2007). Sylvatic *Triatoma infestans* (Reduviidae, Triatominae) in the Andean valleys of Bolivia.. Acta Trop.

[14] Ceballos LA, Picccinali RV, Berkunsky L, Kitron U, Gurtler RE (2009). First finding of melanic sylvatic *Triatoma infestans* (Hemiptera: Reduviidae) colonies in the Argentine Chaco.. J Med Entomol.

[15] Buitrago R, Waleckx E, Bosseno MF, Zoveda F, Vidaurre P (2010). First report of widespread wild populations of *Triatoma infestans* (Reduviidae, Triatominae) in the valleys of La Paz, Bolivia.. Med Hyg.

[16] Thorne C, Serpell J (1995). Feeding behaviour of domestic dogs and the role of experience.. The Domestic Dog: its Evolution, Behaviour and Interactions with People..

[17] Albone ES (1984). Mammalian semiochemistry: the investigation of chemical signals between mammals.

[18] Browne C, Stafford K, Fordham R (2006). The use of scent-detection dogs.. Ir Vet J.

[19] Hebard C (1993). Use of search and rescue dogs.. J Am Vet Med Assoc.

[20] Engeman RM, Vice DS, York D, Gruver KS (2002). Sustained elevation of the effectiveness of detector dogs for locating brown tree snakes in cargo outbound from Guam.. Int Biodeterior Biodegrad.

[21] Nakash J, Osem Y, Kehat M (2000). A suggestion to use dogs for detecting red palm weevil (Rhynchophorus ferrugineus) infestation in date palms in Israel.. Phytoparasitica.

[22] Browne CM (2005). The Use of Dogs to Detect New Zealand Reptile Scents..

[23] Wallner WE, Ellis TL (1976). Olfactory detection of gypsy moth pheromone and egg masses by domestic canines.. Environ Entomol.

[24] Brooks SE, Oi FM, Koehler PG (2003). Ability of canine termite detectors to locate live termites and discriminate them from non-termite material.. J Econ Entomol.

[25] Welch JB (1990). A detector dog for screwworms (Diptera: Calliphoridae).. J Econ Entolol.

[26] Shelby RA, Schrader KK, Tucker A, Klesius PH, Myers LJ (2004). Detection of catfish off-flavour compounds by trained dogs.. Aquacult Res.

[27] Wasser SK, Davenport B, Ramage ER, Hunt KE, Parker M (2004). Scat detection dogs in wildlife research and management: application to grizzly and black bears in the Yellowhead Ecosystem, Alberta, Canada.. Can J Zool.

[28] Kauhanen E, Harri M, Nevalainen A, Nevalainen T (2002). Validity of detection of microbial growth in buildings by trained dogs.. Environ Int.

[29] Manrique G, Vitta ACR, Ferreira RA, Zani CL, Unelius R (2006). Chemical communication in Chagas disease vectors. Source, identity and potential function of volatiles released by the metasternal and Brindley's glands of *Triatoma infestans* Adults.. J Chem Ecol.

[30] Vitta CR, Bohman B, Unelius CR (2009). Behavioral and Electrophysiological Responses of *Triatoma brasiliensis* Males to volatiles produced in the Metasternal Glands of Females.. J Chem Ecol.

[31] Lent H, Wygodzinsky P (1979). Revision of the triatominae (Hemiptera, Reduviidae), and their significance as vectors of Chaga's disease.. Bull Am Mus Nat Hist.

[32] Pires HHR, Lorenzo MG, Diotaiuti, Lazarri, Lorenzo Figueiras AN (2002). Aggregation behaviour in *Panstrongylus megistus* and *Triatoma infestans*: inter and intraspecific responses.. Acta Trop.

[33] Rojas JC (1991). Feromonas de agregación y conducta de apareamiento en algunas especies mexicanas en *Triatoma mazzottii* Usinger (Hemiptera: Reduviidae).. Bol Soc Mex Entomol.

[34] Cruz-López L, Malo EA, Rojas JC (1993). Aggregation pheromone in five species of Triatominae (Hemiptera: Reduviidae).. Mem Inst Oswaldo Cruz.

[35] Schofield CJ, Patterson JW (1977). Assembly pheromone of Triatoma infestans and *Rhodnius prolixus* nymphs.. J Med Entomol.

[36] Lorenzo Figueiras AN, Kenigsten A, Lazzari CR (1994). Aggregation in the haematophagous bug *Triatoma infestans*: Chemical signals and temporal pattern.. J Insect Physiol.

[37] Cruz-López L, Malo EA, Rojas JC, Morgan ED (2001). Chemical Ecology of triatominae bugs: vectors of Chagas disease.. Med Vet Entomol.

[38] Cruz-López L, Morgan ED (1995). Chemical investigations of aggregation behaviour of Triatoma bugs (Hemiptera: Reduviidae).. J Chem Ecol.

[39] CIOMS (1985). Council for International Organizations of Medical Sciences.. http://www.cioms.ch/publications/guidelines/1985_texts_of_guidelines.htm.

[40] Spichiger R, Calenge C, Bise B (2004). Geographical zonation in the Neotropics of tree species characteristic of the Paraguay-Paraná Basin.. J Biogeogr.

[41] Mereles F, Pérez de Molas L (2009). *Bulnesia sarmientoi Lorentz ex Griseb.*, (Zygophyllaceae): estudio de base para su inclusión en el Apéndice II de la Convención CITES.. World Wild Fund.

[42] Fundación para el Desarrollo Sustentable del Chaco & Latin America and the Caribean, U.S. (2005). Agency for International Development..

[43] United States Customs Service [USCS] (1979). U.S. Customs narcotics detector dog training..

[44] Garcia AL, Carrasco HJ, Schofield CJ, Stothard JR, Frame IA (1998). Random amplification of polymorphic DNA as a tool for taxonomic studies of triatomine bugs (Hemiptera: Reduviidae).. J Med Entomol.

[45] Brenière SF, Taveira B, Bosseno MF, Ordoñez R, Lozano-Kasten F (2003). Preliminary results of random amplification of polymorphic DNA among Triatominae of the phyllosoma complex (Hemiptera, Reduviidae).. Mem Inst Oswaldo Cruz.

[46] Lyman DF, Monteiro FA, Escalante AA, Cordon-Rosales C, Wesson DM (1999). Mitochondrial DNA sequence variation among triatomine vectors of Chagas' disease.. Am J Trop Med Hyg.

[47] Velázquez CJ, Gonzalez G (1959). Aspectos de la enfermedad de Chagas en el Paraguay.. Rev Goiania Med.

[48] Bejarano JFR (1967). Estado selvático de *Triatoma infestans* y otros aspectos a tener en cuenta para la eliminación de la enfermedad de Chagas.. 2das Jorn Entomoepidemiol Arg.

[49] Usinger RL, Wygodzinsky P, Ryckman RE (1966). The biosystematics of Triatominae.. Ann Rev Entomol.

[50] Noireau F, Flores R, Vargas F (1999). Trapping sylvatic Triatominae (Reduviidae) in hollow trees.. Trans Roy Soc Trop Med Hyg.

[51] Noireau F, Flores R, Gutierrez T, Abad-Franch F, Flores E (2000). Natural ecotopes of *Triatoma infestans* dark morph and other sylvatic triatomines in the Bolivian Chaco.. Trans Roy Soc Trop Med Hyg.

[52] Noireau F, Diosque P, Jansen AM (2009). *Trypanosoma cruzi*: Adaptation to its vectors and its hosts.. Vet Res.

[53] Noireau F, Cortez MGR, Monteiro FA, Jansen AM, Torrico F (2005). Can wild *Triatoma infestans* foci in Bolivia jeopardize Chagas disease control efforts?. Trends Parasitol.

[54] Rojas de Arias A, Guillen I, Inchaustti A, Maldonado M, Samudio M (1993). Prevalence of Chagas disease in Ayoreo communities of the Paraguayan Chaco.. Tropenmed Parasitol.

[55] Rojas de Arias A (2003). Control y Vigilancia de Chagas con la participación comunitaria de etnias indígenas: Una perspectiva de la situación..

[56] Cecere C, Castañera MB, Canale DM, Chuit R, Gürtler RE (1999). *Trypanosoma cruzi* infection in *Triatoma infestans* and other triatomines: long-term effects of a control program in rural northwestern Argentina.. Pan Am J Public Health.

[57] Gonzalez N, Vega C, Rolon M, Rojas de Arias A (2000). Triatomineos y otros artrópodos en nidos de aves de comunidades indígenas y menonitas del Chaco Paraguayo.. XVth Int Congr Trop Med Mal.

[58] Monteiro FA, Barrett TV, Fitzpatrick S, Cordon-Rosales C, Feliciangeli D (2003). Molecular phylogeography of the Amazonian Chagas disease vectors *Rhodnius prolixus* and *R. robustus*.. Mol Ecol.

